# Soil-transmitted helminth infection in pregnancy and long-term child neurocognitive and behavioral development: A prospective mother-child cohort in Benin

**DOI:** 10.1371/journal.pntd.0009260

**Published:** 2021-03-19

**Authors:** Amanda Garrison, Michael Boivin, Babak Khoshnood, David Courtin, Jules Alao, Michael Mireku, Moudachirou Ibikounle, Achille Massougbodji, Michel Cot, Florence Bodeau-Livinec

**Affiliations:** 1 Département Méthodes Quantitatives en Santé Publique (METIS), Ecole des Hautes Etudes en Santé Publique, Rennes, France; 2 Université de Paris, Center of Research in Epidemiology and Statistics/CRESS, INSERM, INRA, Paris, France; 3 Sorbonne Universités, Université de Paris, Paris, France; 4 Departments of Psychiatry and Neurology/Ophthalmology, Michigan State University, East Lansing, Michigan, United States of America; 5 Université de Paris, MERIT, IRD, Paris, France; 6 Service de Pédiatrie, CHU de la Mère et de l’Enfant-Lagune de Cotonou, Cotonou, Benin; 7 School of Psychology, University of Lincoln, Lincoln, England; 8 MRC-PHE Centre for Environment and Health, Department of Epidemiology and Biostatistics, School of Public Health, Imperial College London, London, England; 9 Centre de Recherche pour la lutte contre les Maladies Infectieuses Tropicales, Université d’Abomey-Calavi, Cotonou, Benin; 10 Faculté des Sciences de la Santé, Université d’Abomey-Calavi, Cotonou, Benin; North Carolina State University, UNITED STATES

## Abstract

**Background:**

An estimated 30% of women in Sub-Saharan Africa suffer from soil-transmitted helminth infection during pregnancy (SHIP), which has been shown to increase risk of pre-term birth, low birth weight, and maternal anemia. A previous study in Benin found that SHIP was associated with impaired cognitive and gross motor development scores in 635 one-year-old children. The objective of the present study was to follow children prospectively to investigate whether the association between SHIP and child neurocognitive and behavioral development persisted at age six.

**Principal findings:**

Our prospective child cohort included 487 live-born singletons of pregnant women enrolled in the *Malaria in Pregnancy Preventive Alternative Drugs* clinical trial in Allada, Benin. SHIP was assessed at three antenatal visits (ANVs) through collection and testing of stool samples. Neurocognitive and behavioral development was assessed in six-year-old children by trained investigators using the Kaufman Assessment Battery for Children 2^nd^ edition and the parent-reported Strengths and Difficulties Questionnaire (SDQ). Multiple linear regression models generated coefficients and 95% confidence intervals and potential mediating factors were tested. Prevalence of SHIP was 13% at the 1^st^ ANV, 9% at the 2^nd^ ANV, and 1% at delivery. SHIP was not associated with low neurocognitive scores in children at six years. Higher SDQ internalizing scores, indicating increased emotional impairments in children, were associated with helminth infection at the 2^nd^ ANV/delivery 1.07 (95% CI 0.15, 2.00) and at least once during pregnancy 0.79 (95% CI 0.12, 1.46) in adjusted models. Mediation analysis did not reveal significant indirect effects of several mediators on this association.

**Conclusions:**

Our study shows that while SHIP is not associated with impaired long-term neurocognitive development, infections may have significant negative impacts on emotional development in six-year-old children. SHIP remains a critical public health issue, and adequate prevention and treatment protocols should be enforced in low- and middle-income countries.

## Author summary

Soil-transmitted helminth infections impact 1.5 billion individuals, primarily in low- and middle- income countries, each year and contribute to malnutrition, anemia, and impaired neurocognitive development in children. However, these infections in pregnancy and their impact on offspring have been less studied. One previous study found associations between soil-transmitted helminth infection during pregnancy and impaired cognitive functioning in offspring one year after birth. The current study aimed to follow these children prospectively until six years in order to confirm whether these associations persisted or not. Infections during pregnancy were no longer associated with cognitive or motor functioning in children; however, infections were associated with impaired behavioral development. Animal-based models have hypothesized maternal inflammation and poor birth outcomes to be the mechanisms behind this relationship; however, our findings did not support these mechanisms. This is one of very few prospective cohort studies in Sub-Saharan Africa to investigate these associations, and more research is needed to corroborate results. Limitations include limited power and the possibility that results are due to chance from multiple statistical tests. Adequate and accessible prevention and treatment efforts in pregnancy and childhood should be provided to populations in low- and middle- income countries at high risk of infection.

## Introduction

Soil-transmitted helminths infect approximately 1.5 billion people worldwide annually [1], contributing to more than five million disability-adjusted life years (DALYs)[2]. Symptoms of these infections include diarrhea and abdominal pain, and infection can have long-term consequences on development and nutritional status [3]. The term ‘soil-transmitted helminths’ refers to several species of parasitic worms, the most common of which are roundworms, *Ascaris lumbricoides*, whipworms, *Trichuris trichiura*, and hookworms, *Ancylostoma duodenale* and *Necator americanus* [4]. Pregnant women and children are especially vulnerable to hookworm infection, a known risk factor for iron deficiency anemia, since these populations tend to have the lowest iron stores [5,6]. During their adult worm-stage, helminths live in the intestines of infected individuals and transmit their eggs within human feces. In areas with inadequate sanitation and plumbing, human feces may contaminate the soil which is ingested from improperly cleaned or prepared vegetables, contaminated water sources, or direct ingestion by children [7]. Hookworm species infect individuals through penetration of the skin when it is exposed to larvae from hatched eggs in the soil [8]. Developing countries, particularly in Sub-Saharan Africa (SSA), are at higher risk of soil-transmitted helminth infection due to conditions of poverty, poor sanitation, limited resources, and an abundance of the population living in rural areas [9,10].

Prevalence of soil-transmitted helminth infection during pregnancy (SHIP) in SSA is estimated to range from 11–31%, according to previous studies [10–12]. Often, infected individuals in SSA countries display polyparasitism, meaning they are simultaneously infected with more than one species of helminth [13,14]. SHIP is known to have negative consequences on birth and maternal outcomes, such as pre-term birth, low birth weight, and maternal anemia [15,16]. The World Health Organization (WHO) recommends preventive chemotherapy in the form of a single dose of either 400mg of albendazole or 500mg of mebendazole for pregnant women during the first trimester in areas of the world with a high baseline prevalence of helminth infection (20%) and anemia (40%) in pregnant women [17].

Several studies have shown the detrimental impacts of soil-transmitted helminth infection during childhood on child growth and neurocognitive development [18,19]; however, little research has focused on how exposure to these infections during pregnancy may impact child neurocognitive development. Two studies from a birth-cohort in Uganda have shown that prenatal exposure to helminth infection did negatively impact early cognitive functioning in 15-month old infants [20]; however, anti-helminthic treatment did not reverse or impact cognitive development in these offspring in early or late childhood [21]. Evidence from some studies have shown that infections such as maternal Human Immunodeficiency Virus (HIV) and malaria during pregnancy remain associated with poor neurocognition in children even at 16 years of age [22,23], suggesting that infections during pregnancy can have long-term consequences on child development. A recent study in Benin found associations between hookworm infection during pregnancy and poor cognitive and gross motor scores, as measured by the Mullen Scales of Early Learning (MSEL), in children at one year of age [24]. However, we found no literature that explored these associations in school-aged children. Additionally, there is a growing body of literature on the consequences of prenatal infection on behavioral development in offspring [25–27]. We found no evidence of studies investigating how maternal parasitic infection, specifically SHIP, may impact long-term behavioral development in children.

Hookworm infection during pregnancy has been shown to increase systemic inflammation and more specifically increase erythrocyte sedimentation rate (ESR), a general marker of inflammation [28,29]. Rodent models have demonstrated that maternal infection leads to inflammatory immune responses that can alter brain development of the fetus and have long-term neurodevelopment consequences [30,31]. Existing evidence suggests that systemic maternal infection and subsequent inflammation response disrupts placental vasculogenesis and angiogenesis, which construct the materno-fetal interface during pregnancy, and in turn negatively impacts fetal growth and development [32]. We were unable to identify previous studies investigating the role of the maternal immunoinflammatory response from SHIP on long-term child neurocognitive development.

Therefore, the main objectives of our study were to investigate the associations between SHIP and neurocognitive and behavioral development outcomes in a population of children in Benin prospectively at six years of age. Infections from hookworms were analyzed separately from other helminth infections due to the significant susceptibility in this population to these infections and their subsequent consequences on iron stores. A secondary objective was to test the indirect effects of SHIP exposure on development through potential mediating factors identified in the literature to explore previously hypothesized mechanisms. Our hypothesis was that SHIP would remain associated to impaired neurocognitive and behavioral development in this prospective cohort at six years of age, and that this relationship would be mediated by hypothesized factors such as inflammation.

## Methods

### Ethics statement

Ethical approval for all studies mentioned in this article was obtained by the institutional review boards of the Beninese Ethical Committee of the Faculté des Sciences de la Santé (FSS) and the Committee of Ethical Research of the Applied Biomedical Sciences Institute (CER-ISBA) in Benin, New York University in the United States of America, and the Research Institute for Development’s (IRD) Consultative Ethics Committee in France. Written informed consent was obtained by mothers and guardians of children in the presence of a witness, with thumbprints provided for consent if women could not read and/or write.

### Study population

Our prospective cohort of children included 487 live-born singletons of pregnant women enrolled in the *Malaria in Pregnancy Preventive Alternative Drugs* (MiPPAD*)* clinical trial (NCT00811421) comparing the efficacy of sulfadoxine-pyrimethamine and mefloquine, two intermittent preventive treatments of malaria in pregnancy (IPTp). The clinical trial took place in the district of Allada, Benin and recruited 1,005 women in the second trimester of pregnancy (before 29 weeks’ gestation) who attended their first antenatal visit (ANV) located in one of three local health clinics in Sékou, Allada, or Attogon. Information on inclusion and exclusion criteria, and detailed recruitment procedures, of the clinical trial have been explained in a preceding publication [33]. Briefly, inclusion criteria for women were: a permanent residence in the study area, gestational age ≤28 weeks, HIV-negative at recruitment, no known allergies to sulfa or mefloquine drugs, no treatment of malaria using these drugs within four weeks of recruitment, and no history of severe renal, hepatic, psychiatric, or neurological disease. All 863 surviving, eligible singletons born to clinical trial participants were invited to participate in a follow-up study (TOVI, meaning ‘child’ in the local Benin language of Fon) at one year of age. At one year, 635 of these children were assessed for neurocognitive development, blood samples were taken, and demographic information was collected through questionnaires given to mothers [24]. Finally, 487 children from the birth cohort were followed-up prospectively at six years of age and assessed for neurocognitive and behavioral development within the *Lead and Manganese Exposure and Child Risk* (French acronym: EXPLORE) study.

### Soil-transmitted helminth infection during pregnancy

The main exposure of interest in our study was SHIP, which was assessed during a nested-cohort study within the MiPPAD clinical trial, entitled *Anemia in Pregnancy*: *Etiologies and Consequences* (APEC) [34]. During follow-up, women were seen at a local health clinic on three occasions: the 1^st^ ANV at recruitment, the 2^nd^ ANV at least one month later, and at delivery. As part of the Benin ANV package, women were given malaria IPTp and iron and folic acid supplements at each visit, as well as 600 mg of mebendazole (100 mg twice daily for three days) at the 1^st^ ANV, and again at the 2^nd^ ANV if women tested positive for SHIP. Intake of mebendazole was not directly monitored by health professionals. Women also received insecticide-treated bed nets at recruitment. Containers were given to women at ANVs in order to collect stool samples the following morning to test for infection. Stool samples were also collected 15 days prior to the expected date of delivery, where possible, or within one week after delivery.

Thick smears of stool samples were prepared and assessed using the Kato-Katz technique as outlined by the WHO [35]. Each of the two slides prepared from each sample was systematically and independently examined by two laboratory technicians under a microscope and the mean of the two results was calculated. The standardized fecal egg count (FEC), expressed in eggs per gram (epg), for each stool sample was obtained by counting the number of eggs for each species and multiplying this number by 24. Helminth infection in this article is defined as the presence of at least one egg of any soil-transmitted helminth species found in the stool. Hookworm infection refers to infections caused by *A*. *duodenale* and *N*. *americanus* species. Analyses considered helminth and hookworm infection diagnosed at the 1^st^ ANV, the 2^nd^ ANV/delivery, and at least once during pregnancy. Helminth and hookworm infections diagnosed at the 2^nd^ ANV and at delivery were considered together, with women being considered as infected if found to be positive at either visit; we hypothesized that infections during the third trimester would have similar effects on neurocognitive and behavioral development in children.

### Child development at six years

The main outcomes considered in this study, child neurocognitive and behavioral development, were assessed at six years of age within the EXPLORE study, whose principal objective was to study the impact of lead and manganese exposure on neurocognitive development and growth in children. Participating mothers and their children visited one of two health centers, in Attogon or Sékou, where trained investigators obtained written consent from caregivers and subsequently administered questionnaires to mothers, collected blood and stool samples from children, and assessed them for neurocognitive and behavioral development. Child stool samples were collected in order to test for helminth infection. If a child was ill or had fever when they arrived at the clinic, blood samples were taken to determine presence of infection and children were asked to return at a later date for neurocognitive assessments. Children who were positively diagnosed with malaria, anemia, or helminth infection were treated according to existing national treatment guidelines at the time.

Neurocognitive development in children was assessed using several tests, including the Kaufman Assessment Battery for Children 2^nd^ edition (KABC-II), a psychological diagnostic tool used to assess processing and cognitive ability, as well as planning and learning capabilities, in children. The KABC-II was available in French and for the purposes of this study was translated into the local language of Benin (Fon) by trained investigators. The construct validity of the KABC-II for use in Benin was verified during a pilot study within this population [36]. For the purposes of this study the Mental Processing Index (MPI), a measure of global cognitive ability generated by the KABC-II, was studied. The Bruininks-Oseretsky Test of Motor Proficiency Edition 2 (BOT-2) was administered to evaluate fine and gross motor skills in children. Final composite scores for fine and gross motor skills were standardized by age and sex and used in analyses. Finally, the Test of Variables of Attention (TOVA), a non-language-based performance test, was administered to identify attention deficits and hyperactivity disorders in children. The final score identifying Attention Deficit and Hyperactivity Disorder (ADHD) is calculated based on response times, variability of response times, and rate of deterioration of responses to computerized stimuli appearing on a screen in front of children during the test. Based on United States norms, scores below -1.80 are considered suggestive of ADHD, with higher scores indicating normal attention rates [37]. The TOVA and BOT-2 were not validated for use in this population; however, they have been validated within other populations of children in SSA countries [37]. For the purposes of our analyses, results within our population were not compared to Western norms and instead analyses focused solely on internal comparisons of scores among children.

Behavioral development of children was assessed through the parent-reported Strengths and Difficulties Questionnaire (SDQ) given to mothers [38]. The questionnaire screens for issues related to five subscales: emotional problems, conduct problems, hyperactivity, peer problems, and pro-social behaviors; with the four former subscales used to generate an overall difficulties score. The SDQ was translated into Fon by trained investigators for the purposes of this study. The SDQ further differentiates between emotional and behavioral conduct problems in children through externalizing and internalizing scores generated by its subscales. Scores from the conduct problems and hyperactivity subscales are combined to create the externalizing (behavioral issues) score, while emotional and peer problems are combined to form the internalizing (emotional issues) score. Higher SDQ scores indicate more behavioral and/or emotional issues in children. Due to the fact that illiteracy rate among mothers in this population was 75%, all assessment instructions and questionnaires were spoken aloud to children and caregivers by trained investigators. A common translation of oral instructions from French to Fon was validated by trained assessors and study nurses prior to the onset of assessments.

### Demographic factors

Family socio-demographic information, such as maternal age, socioeconomic status, education level, and pre-pregnancy Body Mass Index (BMI) was collected from women at recruitment during the 1^st^ ANV of the clinical trial. Malaria incidence during pregnancy, diagnosed using the gold standard thick blood smear test, was recorded in women during the trial. Infant sex, gestational age according to fundal height, and birth weight in grams were recorded at delivery. At the time of neurocognitive and behavioral assessments in children at six years, questionnaires were given to mothers to collect additional demographic information. A continuous score was generated for family possessions to indicate socioeconomic status; with scores given to items owned by the family of pregnant women such as electricity, a car, a motorcycle, a television, a radio, a bicycle, or cows. Parent-child interactions in the home were assessed using the Home Observation for Measurement of the Environment (HOME) test, which was adapted for use in Benin during a pilot study [39].

Factors included in mediation analyses were inflammation during pregnancy, weight-for-age z-scores in children at six years, gestational age at birth, and birth weight. Inflammation during pregnancy was defined as a C-reactive Protein (CRP) concentration higher than 5 mg/mL at one or more ANVs. Weight-for-age z-scores were calculated according to the WHO z-score classification system to represent the latent variable of child nutrition.

### Statistical analysis

Baseline characteristics and exposure to SHIP within our population followed up six years post-partum were described and compared to those lost to follow-up since birth using the non-parametric Fisher exact test for categorical variables. Risk factors of helminth infection in pregnancy and impaired child neurocognitive and behavioral development were identified within the literature and direct acyclic graphs (DAGs). Adjusted models controlled for maternal age, education, pre-pregnancy BMI, family possession score, HOME score, child sex, child age at assessment, and incidence of malaria during pregnancy. Maternal age, family possession score, HOME score, and child age at assessment were continuous variables in regression models. Maternal education (some/none), child sex (male/female), and incidence of malaria during pregnancy (yes/no) were binary variables. Pre-pregnancy BMI was categorized as underweight (<18.5), normal (18.5–24.9), or overweight/obese (≥25). Multiple linear regression models were used to test associations between SHIP and hookworm density in pregnancy and child neurocognitive and behavioral development at six years. The variance inflation factor (VIF) was used to detect multicollinearity between co-variates, if present.

For the secondary objective, mediation analysis using Structural Equation Modeling (SEM) further explored mechanisms of significant associations from multiple linear regressions. The total effects of SHIP on child emotional development were explained through the direct effect of the exposure on the outcome and the indirect effects (i.e. effects mediated by potential intermediate factors identified in the literature) of this association [40]. Mediation analyses adjusted for confounding factors included in regression models and bootstrapped, or bias-corrected, confidence intervals (CIs) are displayed.

Several sensitivity analyses were carried out to assess the robustness of analyses. Characteristics of the population analyzed were compared to those of the mothers and children lost to follow-up since birth. Linear regression models between SHIP and MSEL scores of the 487 children from EXPLORE seen at one year were tested to confirm results from the previous study conducted in this population. Multiple linear regression models tested for associations between helminth infection in children at six years and neurocognitive and behavioral development scores. Multiple imputation of missing variables, including outcome variables and confounding factors, and living mother-child pairs lost to follow-up between birth and six years (n = 320) was completed to minimize plausible selection bias. Blood lead level in children and history of hospitalization between one- and six-year follow-ups were tested in additional models for significance.

All statistical analyses were completed using STATA 13.1 (StataCorp. 2013. *Stata Statistical Software*: *Release 13*. College Station, TX: StataCorp LP). Statistical tests were two-tailed with an alpha risk of 5%. Supporting files contain relevant information regarding the epidemiological conduct of this paper (S1 STROBE Checklist) and data used in analyses (S1 Data).

## Results

**Fig 1:**
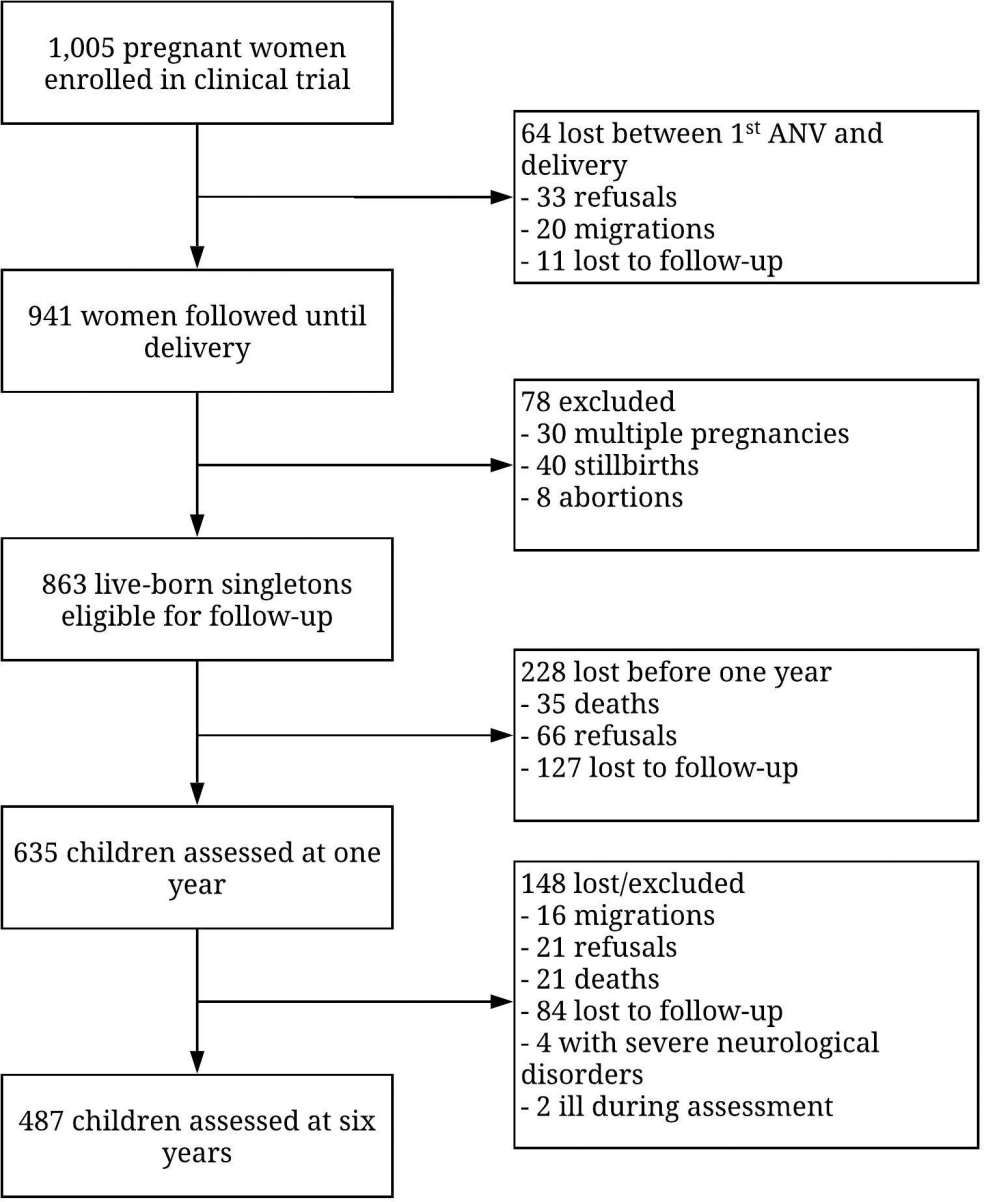
Population flowchart from second trimester of pregnancy until child follow-up at six years of age.

Of the 863 singletons born alive to mothers in the MiPPAD clinical trial, 487 (61%) of eligible children were assessed at 6 years of age and included in our analyses (Fig 1). Four children with severe neurological and mental disorders were excluded from analyses, as well as two children who presented with fever (temperature ≥ 37.5°C) at the time of neurodevelopment assessments.

Table 1 shows the comparison of maternal and infant demographic characteristics at birth and maternal exposure to helminths during pregnancy between children included and excluded in analyses. Mother-child pairs included in our analyses were of higher maternal age, more likely to be multigravida, and of higher parental education levels.

**Table 1:** Demographic and exposure characteristics of mothers and children included in analyses compared to those lost to follow-up.

	*Included*	*Lost*	
	(N = 487)	(N = 320)	p-value^1^
	n (%)	n (%)	
*Demographic Characteristics*			
***Maternal age***			0.06
*<25*	196 (40)	147 (46)	
*25–29*	127 (26)	91 (28)	
*30+*	162 (34)	82 (26)	
***Gravidity***			0.001
*Primigravida*	78 (16)	83 (26)	
*Multigravida*	399 (84)	232 (74)	
***Marital status***			0.002
*Not married*	7 (1)	4 (1)	
*Monogamous*	298 (62)	233 (72)	
*Polygamous*	180 (37)	82 (27)	
***Mother education (1 year)***			0.02
*No education*	333 (69)	194 (61)	
*Primary education/+*	152 (31)	125 (39)	
***Father education (1 year)***			0.04
*No education*	251 (52)	141 (44)	
*Primary education/+*	234 (48)	178 (56)	
***Child sex***			0.54
*Female*	242 (50)	167 (52)	
*Male*	242 (50)	153 (48)	
***Birth weight***			0.85
*Low (<2500g)*	17 (3)	12 (4)	
*Normal (≥* *2500g)*	468 (97)	307 (96)	
***Gestational age***			0.43
*Pre-term (<37 weeks)*	16 (3)	14 (4)	
*Term (≥* *37 weeks)*	469 (97)	305 (96)	
*Exposure Characteristics*			
***Helminth infection***			0.32
*Never infected*	396 (82)	270 (84)	
*Infected at least once*	89 (18)	50 (16)	
***Helminth infection***			
*1*^*st*^ *ANV*	59 (12)	33 (10)	0.41
*2*^*nd*^ *ANV/delivery*	42 (13)	24 (12)	0.97
***Hookworm infection***			0.47
*Never infected*	411 (85)	277 (87)	
*Infected at least once*	74 (15)	43 (13)	
***Hookworm infection***			
*1*^*st*^ *ANV*	46 (10)	28 (9)	0.72
*2*^*nd*^ *ANV/delivery*	36 (11)	19 (10)	0.74

1 P-values from Fisher exact tests

Prevalence of helminth infection in pregnant women during follow-up was 12.6% at the 1^st^ ANV, 8.5% at the 2^nd^ ANV, and 1.2% at delivery (Table 2). The majority of infections in pregnancy were not chronic, with 15% of infected women having more than one infection over the course of her pregnancy. The proportion of infections at each visit caused by hookworm species was 77.9%, 85%, and 100%, respectively. The prevalence of helminth infection in children at six years was 44.6%; 63.7% of infections due solely to hookworms, 27.4% due solely to roundworm, 8.2% due to polyparasitism of hookworm and roundworm species, and <1% due to tapeworm.

**Table 2:** Description of infection in women during pregnancy and in children at six years.

*Parameter*	N	n (%)
***Helminth Infection***		
*1*^*st*^ *ANV*	480	58 (12)
*2*^*nd*^ *ANV*	472	40 (9)
*Delivery*	334	4 (1)
***Helminth species at 1***^***st***^ ***ANV***	58	
*Hookworms*		45 (78)
*Ascaris lumbricoides*		7 (12)
*Trichuris trichiura*		5 (9)
*Other*		1 (2)
***Helminth species at 2***^***nd***^ ***ANV***	40	
*Hookworms*		34 (85)
*Ascaris lumbricoides*		4 (10)
*Trichuris trichiura*		3 (8)
*Other*		2 (5)
***Helminth species at delivery***	4	
*Hookworms*		4 (100)
*Ascaris lumbricoides*		--
*Trichuris trichiura*		--
*Other*		--
***Hookworm density***^***1***^^,^^***2***^	73	
*1*^*st*^ *ANV*		72 (24–984)
*2*^*nd*^ *ANV*		72 (24–600)
*Delivery*		264 (72–624)
***Number of helminth infections during pregnancy***	487	
*None*		399 (82)
*1 infection*		74 (15)
*2 infections*		14 (3)
***Co-infection with malaria during pregnancy***	487	
*1*^*st*^ *ANV*		8 (2)
*2*^*nd*^ *ANV*		--
*Delivery*		1 (<1)
***Helminth infection in children at six years***	327	
*No infection*		181 (55)
*Infection*		146 (45)
***Helminth species in children at six years***	146	
*Hookworms*		93 (64)
*Ascaris lumbricoides*		40 (27)
*Trichuris trichiura*		--
*Strongyloides stercoralis*		--
*Enterobius vermicularis* *Hookworm/lumbricoides* *co-infection*		-- 12 (8)
*Taenia*		1 (<1)

1 Hookworm density in eggs per gram (epg) after multiplication factor

2 Median (range)

Crude and adjusted multiple linear regression models tested the associations between helminth infection and hookworm density in women during pregnancy and neurocognitive development outcomes in their children at six years of age (Table 3). Adjusted models were controlled for maternal age, education, pre-pregnancy BMI, family possession score, HOME score, child sex, age at the time of assessment, and malaria incidence during pregnancy. Helminth infection diagnosed in pregnant women at each ANV and overall helminth infection and hookworm density during pregnancy were not found to be associated with neurocognitive development, motor function, or attention/hyperactivity disorder in children at six years of age measured by the KABC-II, BOT-2, and TOVA tests, respectively. Helminth infection in children at the time of neurocognitive assessments was also not found to significantly impair test scores.

**Table 3:** Multiple linear regression models between SHIP and child neurocognitive development scores at six years.

	*KABC-II Mental Processing Index*		*BOT-2 Motor Composite Score*		*TOVA ADHD Score*	
	Coefficient (95% CI)		Coefficient (95% CI)		Coefficient (95% CI)	
	Crude	Adjusted^1^	Crude	Adjusted^1^	Crude	Adjusted^1^
	N = 478	N = 456	N = 474	N = 452	N = 442	N = 422
***Helminth infection at 1***^***st***^ ***ANV***	-0.09 (-3.46, 3.28)	0.71 (-2.51, 3.92)	-1.82 (-4.34, 0.70)	-0.04 (-2.36, 2.27)	-0.48 (-1.29, 0.33)	-0.06 (-0.86, 0.73)
***Helminth infection at 2***^***nd***^ ***ANV/delivery***	-2.65 (-6.57, 1.26)	-2.04 (-5.82, 1.73)	0.22 (-2.72, 3.16)	-0.75 (-1.93, 3.43)	-0.02 (-0.95, 0.91)	0.02 (-0.89, 0.93)
***Helminth infection once in pregnancy***	-1.24 (-4.08, 1.60)	-0.71 (-3.41, 2.00)	-0.85 (-2.97, 1.28)	0.17 (-1.77, 2.11)	-0.27 (-0.95, 0.41)	-0.03 (-0.69, 0.64)
***Hookworm infection at 1***^***st***^ ***ANV***	0.82 (-2.94, 4.59)	1.74 (-1.86, 5.33)	-0.71 (-3.54, 2.12)	1.20 (-1.39, 3.80)	-0.45 (-1.36, 0.45)	-0.01 (-0.91, 0.89)
***Hookworm infection at 2***^***nd***^ ***ANV/delivery***	-3.75 (-7.96, 0.46)	-2.67 (-6.74, 1.41)	0.24 (-2.92, 3.40)	0.68 (-2.20, 3.57)	0.12 (-0.87, 1.12)	0.15 (-0.82, 1.11)
***Hookworm infection once in pregnancy***	-1.51 (-4.58, 1.55)	-0.78 (-3.70, 2.15)	-0.40 (-2.69 1.89)	0.52 (-1.57, 2.62)	-0.24 (-0.97, 0.49)	-0.03 (-0.74, 0.68)
***Hookworm density during pregnancy***	0.01 (-0.01, 0.02)	0.01 (-0.01, 0.02)	0.01 (-0.01, 0.02)	0.01 (-0.01, 0.02)	-0.01 (-0.01, 0.01)	0.01 (-0.01, 0.01)
	N = 326	N = 307	N = 324	N = 305	N = 303	N = 288
***Child helminth infection at 6 years***	-0.71 (-3.28, 1.87)	-1.05 (-3.60, 1.50)	-1.34 (-3.24, 0.56)	-0.90 (-2.60, 0.79)	0.19 (-0.43, 0.80)	0.21 (-0.39, 0.82)

1 Adjusted for maternal age, education, pre-pregnancy BMI, family possession score, HOME score, child sex, child age at assessment, and incidence of malaria during pregnancy

Table 4 illustrates that SHIP was not associated with a higher total SDQ score in crude or adjusted models. However, SHIP was associated with impaired emotional development as measured by the SDQ internalizing score. Helminth infection at the 1^st^ ANV was associated with higher internalizing scores 0.82 (95% CI 0.02, 1.62) in the crude model. Helminth infection at the 2^nd^ ANV/delivery was associated with higher internalizing scores in both crude 1.13 (95% CI 0.21, 2.05) and adjusted 1.07 (95% CI 0.15, 2.00) models. Helminth infection at any point in pregnancy was also associated with higher internalizing scores in crude 0.78 (95% CI 0.78, 1.45) and adjusted 0.79 (95% CI 0.12, 1.46) models. Infection did not seem to significantly impair behavioral development measured by the SDQ externalizing scores. Hookworm density during pregnancy and helminth infection in children at the time of assessment were not found to be associated with SDQ scores.

**Table 4:** Multiple linear regression models between SHIP and child behavioral and emotional development scores at six years.

	*Strengths & Difficulties*		*Strengths & Difficulties*		*Strengths & Difficulties*	
	Total Score		Internalizing Score		Externalizing Score	
	Coefficient (95% CI)		Coefficient (95% CI)		Coefficient (95% CI)	
	Crude	Adjusted^1^	Crude	Adjusted^1^	Crude	Adjusted^1^
	N = 479	N = 458	N = 479	N = 458	N = 479	N = 458
***Helminth infection at 1***^***st***^ ***ANV***	0.83 (-0.67, 2.33)	1.02 (-0.52, 2.55)	0.82 (0.02, 1.62) *	0.77 (-0.04, 1.58)	0.01 (-1.00, 1.01)	-0.25 (-0.77, 1.26)
***Helminth infection at 2***^***nd***^ ***ANV/delivery***	1.68 (-0.07, 3.42)	1.45 (-0.32, 3.22)	1.13 (0.21, 2.05) *	1.07 (0.15, 2.00) *	0.55 (-0.62, 1.71)	0.38 (-0.80, 1.55)
***Helminth infection once in pregnancy***	0.81 (-0.45, 2.07)	0.88 (-0.40, 2.16)	0.78 (0.11, 1.45) *	0.79 (0.12, 1.46) *	0.03 (-0.81, 0.87)	0.09 (-0.76, 0.94)
***Hookworm infection at 1***^***st***^ ***ANV***	0.63 (-1.05, 2.31)	0.80 (-0.93, 2.52)	0.65 (-0.24, 1.55)	0.65 (-0.25, 1.56)	-0.02 (-1.14, 1.10)	0.14 (-0.99, 1.27)
***Hookworm infection at 2***^***nd***^ ***ANV/delivery***	1.30 (-0.58, 3.17)	0.87 (-1.05, 2.78)	0.95 (-0.03, 1.94)	0.85 (-0.15, 1.84)	0.34 (-0.91, 1.60)	0.02 (-1.24, 1.29)
***Hookworm infection once in pregnancy***	0.70 (-0.65, 2.06)	0.69 (-0.70, 2.07)	0.70 (-0.02, 1.42)	0.71 (-0.02, 1.43)	-0.01 (-0.91, 0.91)	-0.02 (-0.94, 0.89)
***Hookworm density during pregnancy***	0.01 (-0.01, 0.01)	0.01 (-0.01, 0.01)	0.001 (-0.01, 0.01)	0.01 (-0.01, 0.01)	0.01 (-0.01, 0.01)	-0.01 (-0.01, 0.01)
	N = 326	N = 308	N = 326	N = 308	N = 326	N = 308
***Child helminth infection at 6 years***	-0.37 (-1.53, 0.79)	-0.17 (-1.38, 1.05)	-0.17 (-0.81, 0.47)	-0.08 (-0.74, 0.57)	-0.20 (-0.98, 0.57)	-0.08 (-0.87, 0.71)

1 Adjusted for maternal age, education, pre-pregnancy BMI, family possession score, HOME score, child sex, child age at assessment, and incidence of malaria during pregnancy

* p≤0.05

** p≤0.01

**Fig 2:**
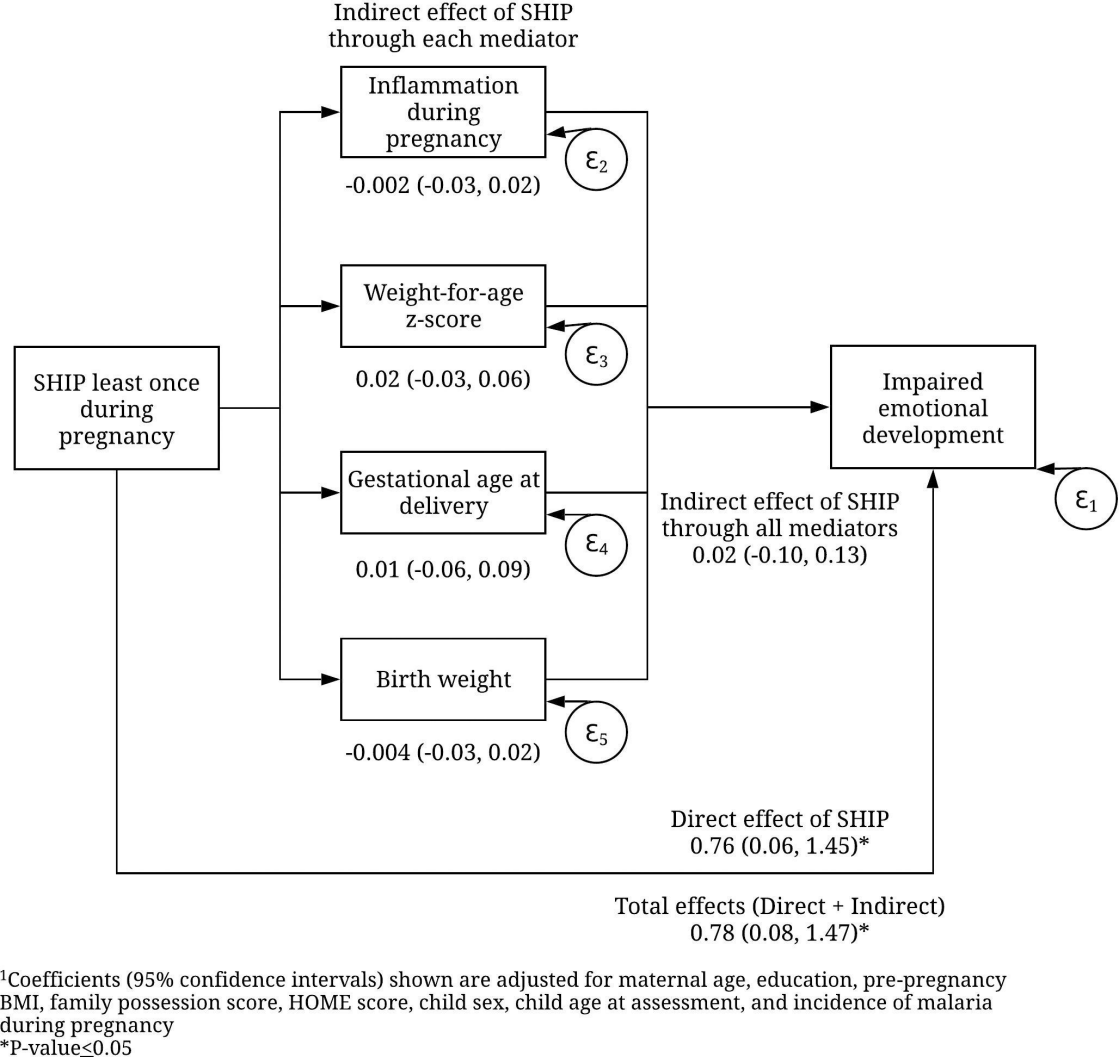
Mediation analysis between SHIP at least once during pregnancy and impaired emotional development in children at six years^1^.

Mediation analyses using SEM methods investigated potential mechanisms behind the significant association seen between SHIP at least once during pregnancy and impaired emotional development as measured by the SDQ internalizing score (Fig 2). Models were adjusted for potential confounding factors used in multiple linear regression and coefficients with 95% confidence intervals are shown. Residuals of mediator and outcome variables are represented by ε_1–5_. Analyses did not reveal significant mediation through the indirect effects of each mediator: inflammation during pregnancy -0.002 (95% CI -0.03, 0.02), child weight-for-age-z-score 0.02 (95% CI -0.03, 0.06), gestational age at delivery 0.01 (95% CI -0.06, 0.09), or birth weight -0.004 (95% CI -0.03, 0.02). The total effect of SHIP on impaired emotional development was statistically significant 0.78 (95% CI 0.08, 1.47), however less than 3% of the total effects could be explained by the indirect effects of potential mediators tested.

After multiple imputation of missing outcomes, co-variables, and children lost to follow-up between birth and six years of age, helminth infections at 1^st^ ANV, 2^nd^ ANV/delivery, and at least once during pregnancy were significantly associated to higher SDQ internalizing scores after adjusting for confounding factors (Table 5). Hookworm infections at the 2nd ANV/delivery and at least once during pregnancy were associated with higher internalizing scores in crude and adjusted models. Blood lead level in children, known to impair long-term neurocognitive development [41,42], was considered in models in sensitivity analyses. Neither blood lead level measured in children at one year nor at six years had a significant impact on the association between SHIP and child development. Hospitalization of children between one and six years was not found to significantly impact associations between SHIP and child development. Analyses between SHIP and MSEL scores at one year were rerun and confirmed previous findings that hookworm infection in pregnancy was associated with impaired cognitive scores in this population.

**Table 5:** Crude and adjusted coefficients (95% CI) from multiple linear regression models after imputation of missing variables and children lost to follow-up.

	*N*	*Neurocognitive development scores*			*Behavioral development scores*		
		*KABC-II Mental Processing Index*	*BOT-2 Motor Composite Score*	*TOVA ADHD Score*	*SDQ Total Score*	*SDQ Internalizing Score*	*SDQ Externalizing Score*
***Helminth infection*** ***at 1***^***st***^ ***ANV***	799	-0.19 (-3.08, 2.70)	-2.02 (-4.70, 0.67)	-0.63 (-1.39, 0.13)	1.19 (-0.28, 2.67)	1.03 (0.24, 1.82) **	0.18 (-0.81, 1.16)
		0.62 (-2.84, 4.07)^1^	-0.85 (-3.44, 1.73)^1^	-0.39 (-1.13, 0.35)^1^	1.16 (-0.34, 2.66)^1^	0.96 (0.16, 1.77)^1^ *	0.21 (-0.79, 1.21)^1^
***Helminth infection*** ***at 2***^***nd***^ ***ANV/delivery***	794	-3.00 (-7.48, 1.48)	0.22 (-2.96, 3.39)	-0.17 (-1.18, 0.84)	1.97 (0.08, 3.86) *	1.28 (0.28, 2.28) **	0.68 (-0.59, 1.96)
		-1.78 (-5.91, 2.35)^1^	1.50 (-1.53, 4.53)^1^	-0.03 (-0.97, 0.92)^1^	1.76 (-0.08, 3.61)^1^	1.26 (0.28, 2.23)^1^ **	0.51 (-0.74, 1.76)^1^
***Helminth infection once in pregnancy***	810	-1.65 (-4.85, 1.55)	-0.91 (-3.30, 1.49)	-0.39 (-1.03, 0.26)	1.06 (-0.19, 2.32)	0.90 (0.24, 1.57) **	0.16 (-0.68, 1.00)
		-0.68 (-3.66, 2.30)^1^	0.09 (-2.18, 2.35)^1^	-0.22 (-0.84, 0.40)^1^	0.96 (-0.30, 2.22)^1^	0.89 (0.22, 1.55)^1^ **	0.08 (-0.76, 0.92)^1^
***Hookworm infection at 1***^***st***^ ***ANV***	799	0.53 (-3.19, 4.24)	-0.89 (-3.79, 2.02)	-0.62 (-1.45, 0.22)	1.02 (-0.70, 2.73)	0.89 (-0.02, 1.81)	0.14 (-1.00, 1.29)
		1.55 (-1.98, 5.09)^1^	0.20 (-2.66, 3.06)^1^	-0.39 (-1.22, 0.44)^1^	0.99 (-0.74, 2.72)^1^	0.84 (-0.08, 1.76)^1^	0.17 (-0.98, 1.32)^1^
***Hookworm infection at 2***^***nd***^ ***ANV/delivery***	794	-3.58 (-8.27, 1.10)	0.14 (-3.22, 3.49)	-0.01 (-1.00, 0.98)	1.62 (-0.42, 3.66)	1.13 (0.05, 2.21) *	0.50 (-0.87, 1.88)
		-2.34 (-6.75, 2.08)^1^	1.06 (-2.17, 4.29)^1^	0.08 (-0.87, 1.02)^1^	1.34 (-0.69, 3.37)^1^	1.13 (0.07, 2.20)^1^ *	0.23 (-1.14, 1.61)^1^
***Hookworm infection once in pregnancy***	810	-1.70 (-4.99, 1.60)	-0.50 (-3.06, 2.07)	-0.36 (-1.02, 0.29)	1.00 (-0.38, 2.37)	0.87 (0.14, 1.61) *	0.14 (-0.78, 1.06)
		-0.71 (-3.86, 2.45)^1^	0.34 (-2.13, 2.81)^1^	-0.23 (-0.87, 0.41)^1^	0.87 (-0.51, 2.26)^1^	0.87 (0.13, 1.60)^1^ *	0.03 (-0.90, 0.96)^1^
***Hookworm density during pregnancy***	119	-0.01 (-0.02, 0.02)	-0.01 (-0.01, 0.01)	-0.01 (-0.01, 0.01)	0.01 (-0.01, 0.01)	0.01 (-0.01, 0.01)	0.01 (-0.02, 0.02)
		-0.01 (-0.04, 0.04)^1^	0.01 (-0.02, 0.03)^1^	-0.01 (-0.01, 0.01)^1^	0.01 (-0.01, 0.02)^1^	0.01 (-0.01, 0.01)^1^	0.01 (-0.01, 0.01)^1^

1 Adjusted for maternal age, education, pre-pregnancy BMI, family possession score, HOME score, child sex, child age at assessment, and incidence of malaria during pregnancy

* p≤0.05

** p≤0.01

## Discussion

This study found that helminth infection during pregnancy was not associated with impaired cognitive ability, gross and fine motor skills, or hyperactivity disorder in children at six years of age. Our analyses did reveal that helminth infection was associated with impaired behavioral development, specifically emotional development in children, after controlling for risk factors. The observed association was not found to be mediated by inflammation during pregnancy, child weight-for-age z-score, or poor birth outcomes. Multiple imputation of missing variables and children lost to follow-up since birth confirmed that both helminth infection and hookworm infection during pregnancy were associated with impaired emotional development.

Our study is the first to our knowledge to investigate how helminth infection measured at different points during pregnancy may impact long-term child neurocognitive development, and is the first study to investigate how these infections during pregnancy impact behavioral development. There is an overall lack of existing literature investigating this subject because of the difficulty in extensive follow-up of SSA cohorts with advanced laboratory and neurocognitive assessment testing. A strength of this study is the prospective nature of the cohort of mothers and children followed from pregnancy until six years post-partum, which included several biological measurements during pregnancy and child development assessments during the follow-up period. The construct validity of the KABC-II was verified in a pilot study within this population, and the SDQ has been used in several studies taking place in SSA countries [36,43,44]. A quality control of the SDQ test was completed at the Global Health Assessment center in Uganda before its use in this study. On-site investigators were trained by study Principal Investigators (PIs) MB and FBL before administering neurocognitive and behavioral assessments. Another strength of this study was the analysis of potential mediators such as gestational age at birth, birth weight, child growth, and inflammation during pregnancy.

Limitations of this study include the small sample size and insufficient power of analyses to show a meaningful difference in KABC-II scores between comparison groups. Given the mean and standard deviation of the KABC-II, a difference of 2.6 points as observed in our results would correspond to an effect size of 0.21; therefore, our study was underpowered to be able to show such a difference in our analyses. Although all investigators administering assessments and questionnaires received the same training and a universal oral translation of instructions was agreed upon, there is still the potential for measurement error. Also, the auto-evaluation of child behavior by parents using the SDQ could have introduced self-reporting bias into our study. However, this is often a bias from child behavioral assessments as many tools rely on observations from parents or teachers. The BOT-2 and TOVA assessments were not validated for use in this population; however, they have been validated for use in other SSA countries [37]. Another limitation of this study is the use of a multitude of statistical tests to explore associations between SHIP and development outcomes, thus the possibility that significant results seen among SDQ internalizing scores could be due to chance cannot be ruled out. Children lost to follow-up between birth and six years were also found to have differing sociodemographic characteristics, however there was no difference in exposure to helminths during pregnancy between these two groups. While this study did take into account several potential confounding and mediating factors, there is still the possibility that some factors were overlooked. For example, conditions of extreme poverty and low social support provided for parents could influence SHIP and neurocognitive development of children and were not included in analyses. Finally, the MiPPAD clinical trial and the APEC nested cohort study supplied helminth prophylaxis to infected women after diagnosis, thus potentially under-estimating exposure to helminths during pregnancy in our population.

A previous study involving 635 Beninese infants found that children with mothers infected by helminths at the 1^st^ ANV and with mothers who had at least one helminth infection during pregnancy had significantly lower mean gross motor scores and cognitive ability than children with mothers who were not infected [24]. We sought to test whether these children still had lower neurocognitive test scores prospectively at six years of age, however our analyses revealed that helminth infection during pregnancy no longer had an impact on long-term child neurocognitive development. Within sensitivity analyses, we reran the same linear regression analyses conducted in this population of children assessed at one year [24] within the population of 487 children included in this paper. We found the same significant associations between SHIP and cognitive development in these children at one year as those found in the previous publication, thus ruling out the possibility that selection bias is responsible for the lack of significant association between SHIP and long-term neurocognitive development at six years (S1 Table). Helminth infection in childhood has been linked to impaired child development; however, results remain equivocal [19,21]. Additional analyses were conducted within this population to test for associations between helminth infection in children at the time of assessment at six years and impaired development scores; however, no significant associations were revealed. Due to its insignificance in univariate analyses, and the fact that helminth infection in pregnancy and in childhood may share common risk factors, authors did not further evaluate childhood helminth infection in mediation analyses. Within our population, 45% of children were diagnosed with helminth infection at six years, despite the national recommendation in Benin to treat children with anti-helminthic prophylaxis every three months beginning at nine months of age. The high prevalence of hookworm infection in children, as opposed to *Ascaris*, could be attributed to water, sanitation, and hygiene (WASH) interventions that have been shown to significantly impact *Ascaris* infection, but have less impact on hookworm infection due to differing modes of transmission of these helminths [45]. This paper provides evidence that these national guidelines are often not respected due to poor governance of public health systems in low-income countries like Benin.

The importance of timing of maternal infection during pregnancy on risk of impaired birth and child outcomes has been widely questioned. Conclusions from previous studies seem inconsistent, with some studies citing no significant difference in child health outcomes based on infection timing during pregnancy [46,47] and others showing that various infections during the second trimester of pregnancy, as opposed to the third trimester, led to more severe neurocognitive and behavioral impairments in children [27,48,49]. Due to the limited existing research on the impact of SHIP on child neurocognitive development, we were unable to form concrete hypotheses for whether or not timing of infection during pregnancy would have a greater impact on neurocognitive scores in children at six years. While results from analyses at one year showed a trend that earlier helminth and hookworm infections (diagnosed in the 2^nd^ trimester) were associated with lower cognitive and motor scores, we do not see the continuation of this trend in our analyses at six years. Further research within prospective cohorts should investigate the role of the timing of SHIP on child health and development outcomes to make clearer conclusions.

Additionally, our study investigated how SHIP could impact child behavioral development measured by the parent-reported SDQ. The hyperactivity and conduct problem scores are combined to form an externalizing score, indicating external behavioral issues, and the peer and emotional problem scores are used to generate an internalizing score to indicate overall emotional issues. We found that SHIP was associated with higher internalizing scores in children, indicating more emotional development problems in children. Several previous studies have found associations between maternal infection during pregnancy and increased risk of emotional and behavioral problems in children through disruption of blood vessel formation between placenta and fetus that have long-term implications on development [26,32,50]. Maternal infection in pregnancy activates the mothers’ inflammatory immune response; several studies have identified this increase in inflammation as the mechanism that leads to increased risk of behavioral problems and disorders in offspring [51–53]. Rodent models have also shown that increased maternal inflammation during pregnancy can directly increase brain inflammation in the fetus and thus alter fetal brain development [25,52]. One previous study specifically found that elevated markers of inflammation in pregnant women were associated with more internalizing and externalizing symptoms in their offspring later in childhood [54]. Ours is the first study to investigate plausible mechanisms of the association between maternal intestinal worm infection and externalizing and internalizing symptoms in school-aged offspring.

Mediation analysis tested the indirect effects of potential mediators in the association between SHIP and impaired emotional development in children measured by the SDQ. SHIP is a known risk factor for birth outcomes such as pre-term birth [48] and low birth weight [55]. Helminths have also been attributed to stunted growth and wasting in children [2,56]. We examined how these mediators, along with inflammation in pregnancy measured by the presence of CRP, could indirectly affect the significant relationship seen between SHIP and impaired emotional development. However, our analyses did not reveal any significant indirect effects from these mediators. Inflammation within mothers in our population could be the result of several different factors, such as malaria or other maternal infection [57] or increased maternal BMI [58]. Therefore, it is difficult to ascertain whether the cause of inflammation within this population is due primarily to helminth infection in pregnancy. Additionally, CRP was the only marker used to measure inflammation during pregnancy; previous studies have indicated eosinophils, IgE, and other cytokines could be more specific inflammatory markers of helminth infection [28]. Other serum cytokines known to be activated in the inflammatory immune response to helminth infection could be studied in future research to evaluate their role as mediators in this relationship. Sensitivity analyses were conducted to test mediation between hookworm infection and impaired emotional development through inflammation, since hookworm infection in particular has been previously linked to systemic inflammation [28], and similarly no significant results were found. Other potential mediating factors such as maternal anemia and child malnutrition were not considered in this paper. Malnutrition was not included due to the fact that it could be both a potential confounding factor and a mediating factor, and because there are several causes of malnutrition in low-income populations such as this. There are also several causes of anemia in at risk populations, and mechanisms between anemia and long-term child development are still unclear. A separate study exploring this complicated and multi-factorial relationship is currently underway within this population, and therefore was not expanded upon for the purposes of this paper.

This study does not confirm hypotheses that SHIP impacts long-term child neurocognitive development at six years. However, it provides evidence that these infections can influence behavioral development during childhood and beyond. The clinical manifestation of impaired neurocognitive development in SSA populations according to Western-based assessment scores is widely unknown, and studies with comparisons of symptoms within SSA populations are needed. Maternal infection by helminths remains a significant public health challenge in many areas of the world, and efforts to control these infections and understand the full scope of their burden on mother and child health are needed. Additionally, the high prevalence of helminth infection in six-year-old children in this study shows that national prevention guidelines for helminth infection may not be adequately followed and implementation of these guidelines must be strengthened to reduce the burden of these infections in school-aged children. Future research should be done to confirm our findings and further investigate how these parasitic infections can impair child development and the role that maternal inflammation may have on this association.

## Supporting information

S1 STROBE Checklist STROBE Checklist for cohort studies. (DOC)

S1 Data Soil-transmitted helminth infection during pregnancy and child development outcomes. (XLS)

S1 Table Associations between SHIP and MSEL scores among 487 children from EXPLORE at one year of age. (DOCX)
