# Supplementary material for: Soil-transmitted helminth infection in pregnancy and long-term child neurocognitive and behavioral development: A prospective mother-child cohort in Benin
Source: PLoS Negl Trop Dis. 2021 Mar 19;15(3):e0009260. doi: 10.1371/journal.pntd.0009260 (PMC7978343; doi:10.1371/journal.pntd.0009260)
Supplement: S1 Table — (DOCX) [file pntd.0009260.s003.docx]

|  | N | ELC scores | | Gross motor scores | |
| --- | --- | --- | --- | --- | --- |
|  |  | Crude (95% CI) | Adjusted (95% CI)^1^ | Crude (95% CI) | Adjusted (95% CI)^2^ |
| **Helminth infection**  At 1^st^ ANV  At 2^nd^ ANV  At delivery | 460  453  399 | -5.24 (-9.18, -1.29)**  -6.06 (-10.63, -1.49)**  -1.88 (-10.71, 6.95) | -5.66 (-9.50, -1.81)**  -4.96 (-9.41, -0.51)*  -0.78 (-9.35, 7.79) | -3.94 (-7.90, 0.03)*  -2.60 (-7.16, 1.97)  1.63 (-6.94, 10.20) | -3.70 (-7.61, 0.20)  -1.54 (-6.04, 2.96)  2.95 (-5.51, 11.40) |
| **Hookworm infection**  At 1^st^ ANV  At 2^nd^ ANV  At delivery | 460  453  399 | -3.48 (-7.85, 0.90)  -6.31 (-11.18, -1.45)**  -0.74 (-10.03, 8.56) | -4.13 (-8.38, 0.12)  -5.00 (-9.75, -0.25)*  0.30 (-8.72, 9.32) | -4.53 (-8.90, -0.16)*  -2.73 (-7.59, 2.13)  3.46 (-5.57, 12.48) | -4.68 (-8.99, -0.38)*  -1.70 (-6.51, 3.10)  4.97 (-3.92, 13.86) |
| **Helminth infection**  Never (ref)  At least once | 470 | 0  -5.43 (-8.64, -2.23)** | 0  -5.15 (-8.27, -2.04)** | 0  -3.21 (-6.46, 0.04)* | 0  -2.58 (-5.77, 0.61) |
| **Hookworm density**  Not infected (ref)  Moderate (<72^§^)  High (>72) | 470 | 0  -3.55 (-8.37, 1.26)  -7.12 (-12.14, -2.10)** | 0  -2.87 (-7.60, 1.86)  -6.63 (-11.50, -1.77)** | 0  -3.48 (-8.33, 1.38)  -6.50 (-11.55, -1.44)** | 0  -3.27 (-8.08, 1.54)  -3.81 (-10.50, -0.54)* |

^§^72=median density (eggs per gram)

*p-value<0.05

**p-value<0.01

^1^Adjusted for maternal education, child sex, and HOME score

^2^Adjusted for maternal education, child sex, family possession score, gravidity, and HOME score

ELC=Early Learning Composite
